# Human Peptides α-Defensin-1 and -5 Inhibit Pertussis Toxin

**DOI:** 10.3390/toxins13070480

**Published:** 2021-07-11

**Authors:** Carolin Kling, Arto T. Pulliainen, Holger Barth, Katharina Ernst

**Affiliations:** 1 Institute of Pharmacology and Toxicology, Ulm University Medical Center, 89081 Ulm, Germany; carolin.kling@uni-ulm.de (C.K.); holger.barth@uni-ulm.de (H.B.); 2 Institute of Biomedicine, Research Unit for Infection and Immunity, University of Turku, FI-20520 Turku, Finland; arto.pulliainen@utu.fi

**Keywords:** pertussis toxin, defensins, ADP-ribosylating toxin, bacterial AB-type toxin, human peptide, antimicrobial peptide, toxin inhibitor

## Abstract

*Bordetella pertussis* causes the severe childhood disease whooping cough, by releasing several toxins, including pertussis toxin (PT) as a major virulence factor. PT is an AB_5_-type toxin, and consists of the enzymatic A-subunit PTS1 and five B-subunits, which facilitate binding to cells and transport of PTS1 into the cytosol. PTS1 ADP-ribosylates α-subunits of inhibitory G-proteins (Gαi) in the cytosol, which leads to disturbed cAMP signaling. Since PT is crucial for causing severe courses of disease, our aim is to identify new inhibitors against PT, to provide starting points for novel therapeutic approaches. Here, we investigated the effect of human antimicrobial peptides of the defensin family on PT. We demonstrated that PTS1 enzyme activity in vitro was inhibited by α-defensin-1 and -5, but not β-defensin-1. The amount of ADP-ribosylated Gαi was significantly reduced in PT-treated cells, in the presence of α-defensin-1 and -5. Moreover, both α-defensins decreased PT-mediated effects on cAMP signaling in the living cell-based interference in the Gαi-mediated signal transduction (iGIST) assay. Taken together, we identified the human peptides α-defensin-1 and -5 as inhibitors of PT activity, suggesting that these human peptides bear potential for developing novel therapeutic strategies against whooping cough.

## 1. Introduction

As an AB_5_ protein toxin, *Bordetella (B.) pertussis* toxin (PT) consists of an enzyme subunit, the A-protomer PTS1, and five B-subunits forming a holotoxin with a pyramid-like structure, which is secreted by the bacteria [1,2,3]. The B-pentamer facilitates binding to the cell surface via sialoglycoproteins that are present on most cell types [1,4,5,6]. After internalization by endocytosis, PT follows a retrograde intracellular transport through the Golgi to the endoplasmic reticulum (ER) [7]. This retrograde transport is inhibited by brefeldin A (BFA), which disrupts vesicle transport between the ER and Golgi apparatus [7,8]. In the ER, the binding of ATP to the toxin causes destabilization of the interaction between PTS1 and the B-pentamer, which results in the release of PTS1 from the holotoxin [9,10,11]. The released PTS1 is thermally unstable, leading to its unfolding under physiological conditions [12,13,14]. Thereby, it is detected by the ER-associated degradation (ERAD) pathway, and transported from the ER to the cytosol. A lack of lysine residues protects PTS1 from ubiquitination, and thus from proteasomal degradation [15]. The molecular mechanism underlying the membrane transport of PTS1 into the cytosol of the target cells is not well understood. We have recently shown that cellular protein folding helper enzymes play a role in the uptake of PTS1 into the host-cell cytosol [16,17]. 

In the cytosol, PTS1 covalently transfers an ADP-ribose moiety from its co-substrate NAD^+^ onto inhibitory α-subunit G-proteins (Gαi) of G-protein-coupled receptors (GPCRs) [18,19]. ADP-ribosylation inactivates Gαi, which results in disturbed cAMP signaling, because Gαi no longer functions as a negative regulator of adenylate cyclase (AC). The consequences of disturbed GPCR and cAMP signaling are multifaceted, and depend on the cell type. In the early stages of infection, PT inhibits the recruitment of neutrophils, monocytes, and lymphocytes to the lung, and leads to a reduction in pro-inflammatory chemokines and cytokines, as well as to increased bacterial burden in a mouse model [20,21,22].

PT is an important virulence factor for causing the disease whooping cough, which is characterized by long-lasting (ca. 10 weeks) paroxysmal coughing. Patients often suffer from secondary complications, such as vomiting and pneumothorax. In severe cases, pneumonia, encephalopathy, seizures, or apnea occur, which can be life-threatening, especially in newborns and young infants [23]. In 2014, the WHO estimated the number of pertussis cases in children <5 years of age at >24.1 million, with 160,700 deaths worldwide [24]. Despite a high vaccination rate in western countries (e.g., ca. 93% vaccination coverage of children starting school in Germany in 2017; ca. 85% vaccination coverage of infants worldwide in 2019 [25,26]), increasing case numbers have been recorded, reaching an all-time high since introducing vaccination in the 1950s. Therapeutic options to treat pertussis are very limited. Antibiotic therapy eliminates *B. pertussis* bacteria, which is important because it prevents transmission by droplet infection. However, antibiotic treatment has no relieving effect on pertussis symptoms, except if treatment is started up to two weeks after infection, which rarely occurs because, in most cases, the diagnosis is made later [23]. It was demonstrated that PT causes severe and long-lasting inflammation of the airways in a mouse model [27]. *B. pertussis* strains not expressing PT did not cause leukocytosis, which is a hallmark of severe pertussis, or death. These results clearly indicate a pivotal role of PT in causing severe courses of disease, which makes it an attractive target for the development of novel pharmacological strategies [28,29,30]. 

Here, the effect of human antimicrobial peptides of the defensin family on PT was investigated in vitro and in living cells. Defensins are small, cysteine-rich cationic peptides, and they play an important role in innate immunity, because they inactivate bacterial pathogens [31,32,33]. However, it was discovered that some defensins additionally neutralize bacterial protein toxins, such as *Clostridioides difficile* toxins or diphtheria toxin [31,34,35,36]. In the present study, we show that α-defensin-1 and -5 inhibited the enzyme activity of PT in vitro, in a concentration-dependent manner. Moreover, when the cells were treated with PT in the presence of α-defensin-1 or -5, the amount of ADP-ribosylated Gαi was reduced, compared to the cells treated with PT only. An inhibitory effect of α-defensin-1 and -5 was also shown on the PT-mediated effects on cAMP signaling in a living cell-based assay. Notably, the structurally related ß-defensin-1 exhibited no inhibition in the same experiments, suggesting a specific inhibitory mechanism of α-defensin-1 and -5 on PT. 

## 2. Results

### 2.1. Human α-Defensin-1 and -5 Inhibit the Enzyme Activity of PTS1 In Vitro

**Figure 1:**
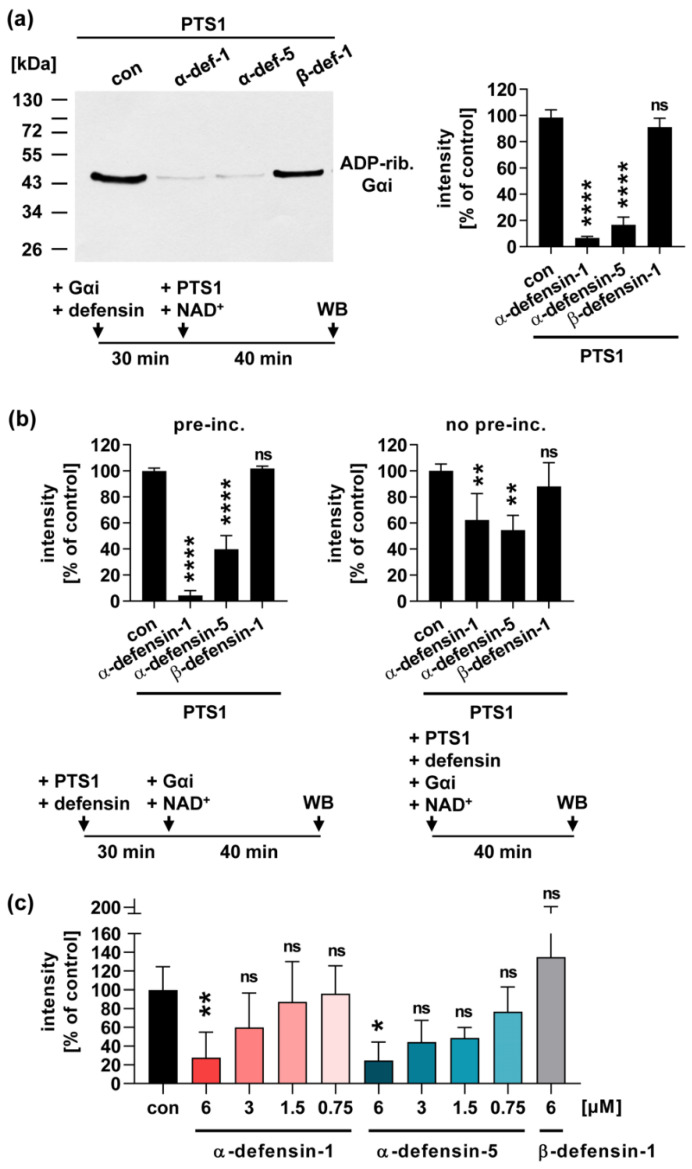

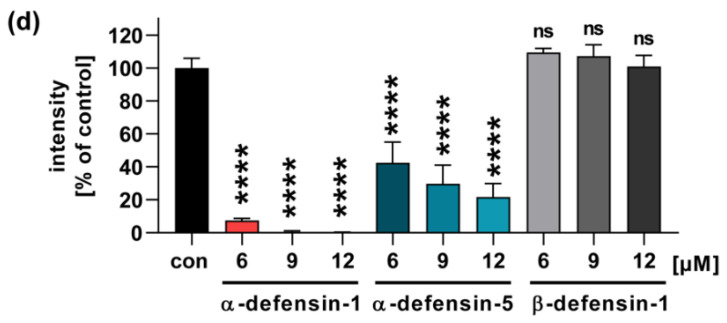
Human α-defensin-1 and -5 inhibit enzyme activity of PTS1. (**a**) Recombinant Gαi was pre-incubated with 6 µM α-defensin-1, α-defensin-5 or ß-defensin-1, or with the respective amount of solvent (H_2_O) for 30 min at 37 °C. PTS1 (50 nM) and biotin-labeled NAD^+^ were added for 40 min at RT. Biotin-labeled, i.e., ADP-ribosylated Gαi, was detected via Western blot. One representative Western blot result is shown, and the bar graph shows quantified Western blot signals from three independent experiments. Values are given as percent of control, mean ± SEM (n = 6 from three independent experiments). (**b**) PTS1 (50 nM) was pre-incubated with 6 µM α-defensin-1, α-defensin-5 or ß-defensin-1, or with the respective amount of solvent (H_2_O) for 30 min at 37 °C. Gαi and biotin-labeled NAD^+^ were added for 40 min at RT. Biotin-labeled, i.e., ADP-ribosylated Gαi was detected via Western blot. Bar graph shows quantified Western blot signals from three independent experiments. Values are given as percent of control, mean ± SD (n = 3 from three independent experiments). (**c**) PTS1 (50 nM) was pre-incubated with decreasing amounts of α-defensin-1 or α-defensin-5, 6 µM ß-defensin-1, or with the respective amount of solvent (H_2_O) for 30 min at 37 °C. Gαi and biotin-labeled NAD^+^ were added for 40 min at RT. Biotin-labeled, i.e., ADP-ribosylated Gαi, was detected via Western blot. Bar graph shows quantified Western blot signals from at least four independent experiments. Values are given as percent of control, mean ± SEM (n = at least four from at least four independent experiments). (**d**) PTS1 (50 nM) was pre-incubated with increasing amounts of α-defensin-1, α-defensin-5 or ß-defensin-1, or with the respective amount of solvent (H_2_O) and then samples were processed as described in (c). (**a–d**) Significance was tested using one-way ANOVA followed by Dunnett’s multiple comparison test and refers to respective untreated controls (* *p* ≤ 0.1, ** *p* ≤ 0.01, **** *p* ≤ 0.0001, *ns* not significant). WB = Western blot.

Prompted by previous findings that defensins inhibited the enzyme activity of ADP-ribosylating toxins such as diphtheria toxin [31], we investigated whether defensins interfere with the enzyme activity of PTS1. Therefore, recombinant PTS1 was incubated with Gαi, in the presence of its biotin-labeled co-substrate NAD^+^. PTS1 transfers the ADP-ribose moiety, and thereby the biotin-label, onto its specific substrate Gαi. ADP-ribosylated, i.e., biotin-labeled Gαi, was detected by Western blot. The results in Figure 1 show that α-defensin-1 and -5, but not ß-defensin-1, inhibit the enzyme activity of PTS1 in vitro. The inhibitory effect was comparable when either Gαi (Figure 1a) or PTS1 (Figure 1b) were pre-incubated with the defensins in a reaction tube. Without pre-incubation, inhibition by α-defensin-1 and -5 was still observed, but to a slightly lesser extent (Figure 1b). Moreover, a concentration dependency of the inhibitory effect of α-defensin-1 and -5 was observed. Concentrations of 6 µM or higher of α-defensin-1 or -5 showed a significant inhibitory effect on PTS1 enzyme activity (Figure 1c,d). 

### 2.2. In the Presence of α-Defensin-1 and -5 Less Gαi Is ADP-Ribosylated in PT-Treated Cells

**Figure 2:**
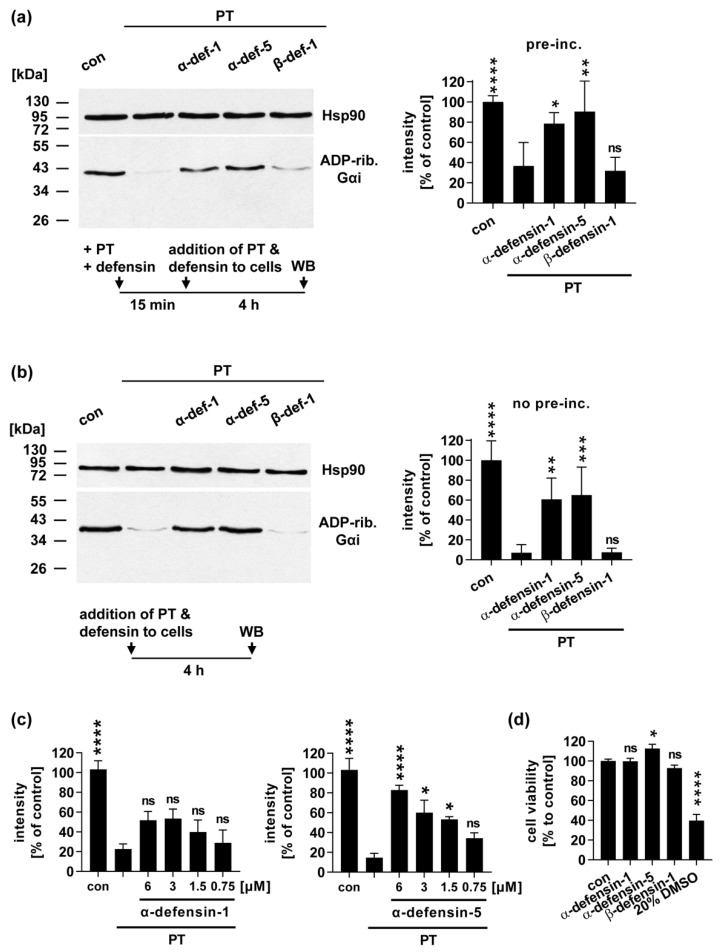
Effect of human α-defensin-1 and -5 on the ADP-ribosylation status of Gαi in PT-treated cells. (**a**) PT (10 ng/mL) was pre-incubated with 6 µM α-defensin-1, α-defensin-5 or ß-defensin-1, or with the respective amount of solvent (H_2_O) for 15 min at room temperature and then added to CHO-K1 cells for 4 h at 37°C. For further control, cells were left untreated. Cells were lysed and Gαi, which has not been ADP-ribosylated in the cells during intoxication, was ADP-ribosylated and thus biotin-labeled by incubation with PTS1 in the presence of biotin-labeled NAD^+^. Biotin-labeled Gαi was detected via Western blot. Comparable protein loading was confirmed with Ponceau-S- (not shown) and Hsp90- staining. One representative Western blot is shown, and bar graphs show quantified Western blot signals from three independent experiments. Values are given as percent of untreated control, normalized to Hsp90, mean ± SD (n = 3 from three independent experiments); (**b**) PT (10 ng/mL) and 6 µM α-defensin-1, α-defensin-5 or ß-defensin-1, or the respective amount of solvent (H_2_O) were added to cells at the same time. Subsequently, the experiment was performed as described in (a); (**c**) CHO-K1 cells were treated at the same time with PT (10 ng/mL) and decreasing concentrations of α-defensin-1 or α-defensin-5 (as indicated) for 4 h at 37 °C. ADP-ribosylation status was determined as described in (a). Bar graphs show quantified Western blot signals from four independent experiments. Values are given as percent of untreated control, normalized to Hsp90, mean ± SEM (n = 4 from four independent experiments); (**d**) CHO-K1 cells were treated with 6 µM α-defensin-1, α-defensin-5 or ß-defensin-1, or with the respective amount of solvent (H_2_O) or 20% DMSO as a positive cell viability decreasing control for 4 h at 37 °C. Cell viability was measured by MTS assay. Absorbance values are given as percent of untreated control, mean ± SEM (n = 12 from four independent experiments). Significance was tested using one-way ANOVA followed by Dunnett’s multiple comparison test and refers to controls treated with PT only (**a**–**c**) or untreated controls (**d**) (* *p* ≤ 0.1, ** *p* ≤ 0.01, *** *p* ≤ 0.001, **** *p* ≤ 0.0001, *ns* not significant). WB = Western blot.

After demonstrating a significant and specific inhibition of PTS1 enzyme activity by α-defensin-1 and -5, we tested whether the defensins also impair intoxication of CHO-K1 cells with the PT holotoxin. PT and the defensins were either added simultaneously to the cells (Figure 2a), or after pre-incubation in a reaction tube (Figure 2b). After 4 h, the ADP-ribosylation status of Gαi was determined in the lysates of treated cells, by sequential ADP-ribosylation. Therefore, recombinant PTS1 and biotin-NAD^+^ were added to the lysates. This allows ADP-ribosylation, and thereby biotin-labeling of Gαi that was not modified during the treatment of the living cells. Thus, the untreated control cells result in a strong signal of biotin-labeled Gαi, and the signal of PT-treated cells is significantly reduced (Figure 2). The samples treated with PT in the presence of α-defensin-1 and -5 resulted in increased signals compared to the samples treated with PT only. This indicates that the cells were protected from intoxication with PT. The inhibition by α-defensin-1 and -5 was comparable with or without the pre-incubation of PT with the defensins. A concentration-dependent inhibition was clearly observed for α-defensin-5, and a trend was revealed for the inhibition by α-defensin-1 (Figure 2c). Again, ß-defensin-1 did not protect the cells from intoxication with PT (Figure 2a and b). Notably, all three defensins did not reduce the cell viability on their own, under the same experimental conditions (Figure 2d). 

### 2.3. α-Defensin-1 and -5 Inhibit the PT-Mediated Effects on cAMP Signaling in Cells

**Figure 3:**
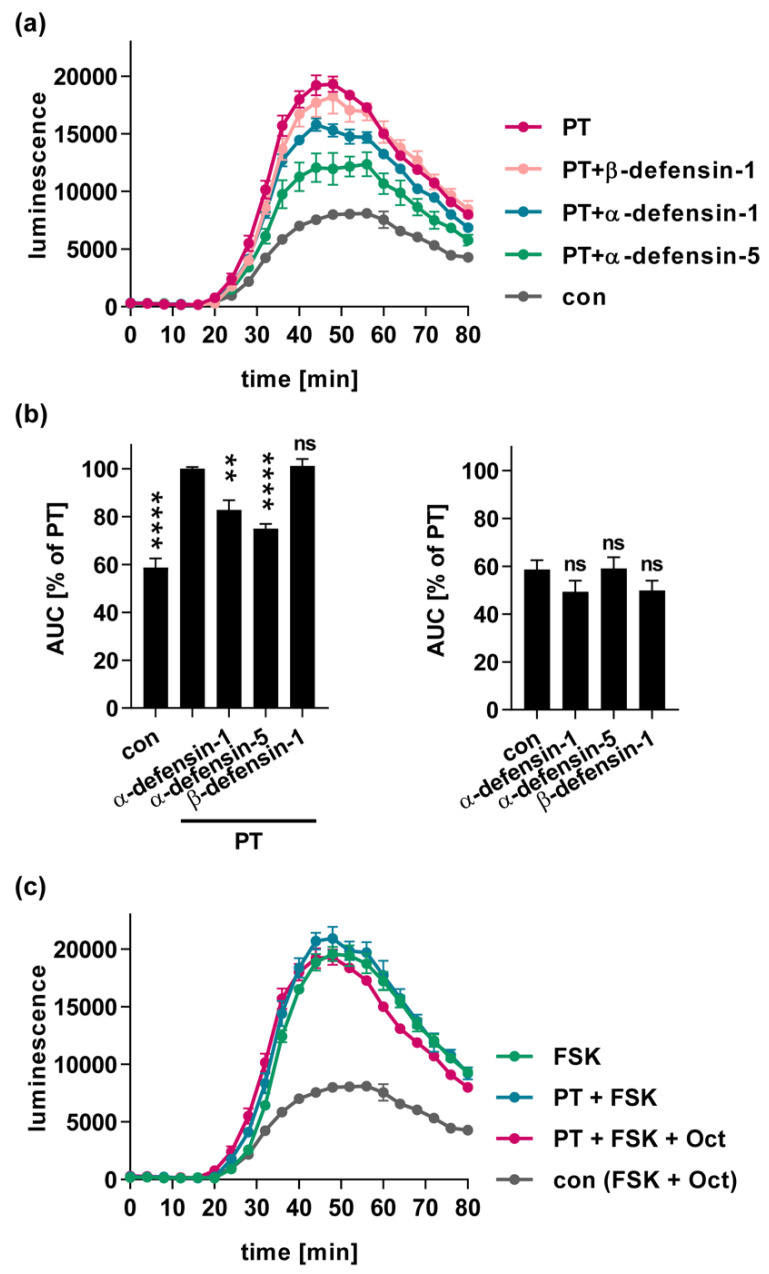
Human α-defensin-1 and -5 inhibit PT-mediated effects on cAMP signaling. PT (100 ng/mL) was pre-incubated with 12 µM α-defensin-1, α-defensin-5 or ß-defensin-1, or with the respective amount of solvent (H_2_O) for 15 min at room temperature and then added to iGIST sensor cells for 5 h at 37°C. For further control, cells were treated only with the solvent of PT. Then, inducing medium containing the luciferase substrate for the luminescent biosensor for cAMP was added. After 15 minutes of baseline measurement, cells were spiked with forskolin to activate adenylate cyclase and octreotide acetate to activate Gαi-coupled SSTR2 GPCR. Luminescence was recorded for further 60 minutes. (**a**) cAMP kinetic curves from one representative experiment are shown as mean ± SD (n = 3 from one experiment), con = cells treated only with forskolin (FSK) plus octreotide (Oct). (**b**) Bar graphs show baseline-subtracted area under the curve (AUC) from at least three independent experiments. Values are given as percent of PT-only-treated samples, mean ± SEM (n = at least nine from at least three independent experiments). Values for control samples are identical in both graphs. Results are shown in two separate graphs for better clarity. Significance was tested using one-way ANOVA followed by Dunnett’s multiple comparison test and refers to samples treated with PT only (left graph) or untreated controls (right graph) (** *p* ≤ 0.01,**** *p* ≤ 0.0001, *ns* not significant). (**c**) For control, iGIST sensor cells were treated only with FSK or with PT plus FSK in the absence of Oct to measure the maximal cAMP response in this assay. Values for control samples and samples treated with PT plus FSK and Oct are identical to values in (**a**).

A consequence of PT-mediated Gαi-ADP-ribosylation is its inactivation, which leads to disturbed cAMP signaling, because Gαi can no longer efficiently inhibit the adenylate cyclase. The iGIST bioassay has been developed to measure the effect of PT on Gαi signaling in cells [37]. The method is based on HEK293 cells that ectopically express the Gαi-coupled GPCR somatostatin receptor 2 (SSTR2), together with a luminescent cAMP probe (iGIST sensor cells). Experimentally, these cells are treated with forskolin, to directly activate the adenylate cyclase, and additionally with octreotide to activate the SSTR2. This only leads to a moderate increase in cAMP in the control samples, because Gαi inhibits the adenylate cyclase due to SSTR2 stimulation (Figure 3a, grey curve). When the cells were treated with PT prior to stimulation with forskolin and octreotide, cAMP levels significantly increased, because inhibition of the adenylate cyclase by Gαi was decreased due to its ADP-ribosylation by PT (Figure 3a, red curve). When the cells were treated with forskolin, but not octreotide, maximal levels of cAMP were reached in the presence and absence of PT (Figure 3c). The treatment of the cells with PT that had been pre-incubated with α-defensin-1 and -5, resulted in an inhibited increase in intracellular cAMP, suggesting that the defensins also impaired PT activity in this bioassay (Figure 3a and b). Notably, ß-defensin-1 did not inhibit the PT-mediated cAMP increase, and in the absence of PT, the defensins had no significant effect on cAMP levels compared to the control samples (Figure 3b, right graph). 

Taken all together, we demonstrated that human α-defensin-1 and -5, but not ß-defensin-1, inhibit the enzyme activity of PTS1 in vitro, and impair the consequences of PT in living cells, as analyzed by the ADP-ribosylation of Gαi, as well as by measuring the PT-mediated cAMP increase in stimulated HEK sensor cells using the iGIST bioassay. The results suggest a potential role for human defensins as inhibitors against PT during *B. pertussis* infection. 

## 3. Discussion

*B. pertussis* produces several virulence factors that contribute to the development of the disease, including the AB_5_-type toxin PT [38]. PT is essential for causing the disease, since *B. pertussis* strains that do not express PT only elicit weak symptoms [28]. The precise role of PT and other virulence factors, as well as their interplay for *B. pertussis* pathogenesis, is not fully understood. It was demonstrated that PT causes severe and long-lasting inflammation of the airways in a mouse model [27]. *B. pertussis* strains not expressing PT did not cause leukocytosis or death. These results clearly indicate a pivotal role of PT in causing severe courses of disease, which makes it an attractive target for the development of novel pharmacological strategies [28,29,30]. 

Here, we demonstrated that the human antimicrobial peptides α-defensin-1 and -5 inhibit the enzyme activity of PT. Moreover, we showed that less Gαi was ADP-ribosylated in the cells treated with PT, and the PT-mediated cAMP increase in the sensor cells was reduced in the presence of α-defensin-1 and -5. Interestingly, these effects were not observed with ß-defensin-1, suggesting that the inhibition of PT by α-defensin-1 and -5 is more specific and not just based on the cationic nature of defensins. Human defensins can be subdivided into α- and ß-defensins based on their molecular weight, and the distribution of cysteines and disulfide bonds [33]. The positive charge of defensins plays an important role in their antimicrobial activity, because this enables the defensins to interact with the negatively charged phospholipids of microbial membranes. Defensins then cause disruption of the membrane, and thereby facilitate the killing of bacteria [33]. So far, it is not fully understood which characteristics and structural elements of defensins are crucial for their anti-toxin activity. Intramolecular disulfide bonds, hydrophobicity, and high arginine content seem to be essential, and dimerization as well as cationicity were important for anti-toxin activity [32]. Interestingly, the arginine content of α-defensin-1 (13.3%) and α-defensin-5 (18.8%) is higher than in ß-defensin-1 (2.7%) [32,39]. Moreover, α-defensin-1 and -5 share a very similar three-dimensional structure that differs from the ß-defensin-1 structure [32,40]. Although the anti-toxin activity cannot be pinpointed to one structural element, these differences between the defensins might be the reason why α-defensin-1 and -5, but not ß-defensin-1, inhibit PT and other toxins. 

Interestingly, it has been reported that, additionally to their antimicrobial activity, human defensins also exhibit anti-toxin activity [32,40]. Both α-defensin-1 and -5 inhibit the *C. difficile* toxins TcdA, TcdB, and CDT. For TcdA and TcdB, the underlying mechanism of inhibition is the aggregation of the toxins by the defensins [34,35,36]. For CDT, it was shown that pore formation by the B-component of the toxin is impaired by α-defensin-1 and -5 [34,35]. A comparable mechanism was observed for the *Clostridium perfringens* iota toxin that is closely related to CDT [41]. Notably, no inhibition of the *C. difficile* toxins or iota toxin was detected for ß-defensin-1 [34,35,41]. Human α-defensin-1 also inhibited the enzyme activity of the *Bacillus anthracis* lethal toxin, a metalloprotease, as well as of the diphtheria toxin and *Pseudomonas* exotoxin ETA, which are both ADP-ribosyltransferases, similarly to PT [31,42]. The active site, in particular the NAD^+^ binding pocket, is structurally conserved among ADP-ribosylating toxins. Thus, the inhibition of ADP-ribosyltransferase activity by α-defensin-1 and -5 might be a common mechanism for this group of toxins. However, further inhibitory mechanisms of defensins against bacterial toxins have been identified, which might also play an additional role for the inhibition of PT by α-defensin-1 and -5. It was shown that the thermally unstable effector domains of MARTX from *V. cholerae* and *Aeromonas hydrophilia*, which display an auto-processing protease and actin crosslinking activity, were inhibited by α-defensin-1 [39]. This inhibition was due to local unfolding, which increased the thermal melting and precipitation, and exposed additional regions for proteolysis [39]. Since the enzyme subunit of PT is also known to be thermally unstable, which is a necessary requisite for its transport from the ER to the cytosol [14], increased unfolding, mediated by the defensins, might be an additional inhibitory mechanism. Up to now, a common mechanism of the inhibition of bacterial toxins has not been identified, and it is not known which is the common characteristic of the small peptides that is essential for inhibition. 

Further, α-defensin-1 is expressed in neutrophils and stored in granules. Upon pro-inflammatory or bacterial stimuli, it is released by degranulation [43]. In the early stages of infection, PT suppresses the recruitment of neutrophils. Later, neutrophils are recruited to the infection site again, but are inhibited by another toxin of *B. pertussis*, the adenylate cyclase toxin (ACT) [44]. Also, α-defensin-5 is found in Paneth cells in the crypts of the small intestine [45], raising the question of its physiological role during the *B. pertussis* infection that is located in the respiratory tract. However, human defensins bear the potential to serve as starting points for the development of novel therapeutic strategies against the severe symptoms of whooping cough, which are associated with PT activity [28,30]. 

## 4. Materials and Methods

### 4.1. Compounds and Reagents

Recombinant Gαi and recombinant PTS1 were expressed and purified as described earlier [46]. The used peptides α-defensin-1 and -5 as well as β-defensin-1 were purchased from PeptaNova (Sandhausen, Germany). *Bordetella pertussis* toxin (PT) was purchased from Sigma-Aldrich, Merck (Darmstadt, Germany).

### 4.2. In vitro Enzyme Activity of PTS1

Recombinant Gαi (0.5 µM) was incubated with recombinant PTS1 (50 nM = 1.17 µg/mL) and biotin-labeled NAD^+^ (1 µM; R&D Systems, Abingdon, United Kingdom) in a 50 nM sodium phosphate buffer (pH 7.0) in the presence of the respective defensin or H_2_O (solvent of defensins) as a control for 40 min at room temperature. Subsequently, samples were subjected to SDS-PAGE and Western blotting with streptavidin–peroxidase (Strep–POD, Sigma-Aldrich, Merck, Darmstadt, Germany) for detection of biotin-labeled and thus ADP-ribosylated Gαi. Densitometric quantification of signals was performed using ImageJ software (NIH). Ponceau-S-staining was used to control equal loading of samples. 

### 4.3. Cell Lines

All materials for cell culture were purchased from Gibco unless indicated otherwise. Chinese hamster ovary cells strain K1 (CHO-K1, DSMZ, Braunschweig, Germany) were cultivated in DMEM and HAM’s F12 (1:1) supplemented with 5% heat-inactivated fetal calf serum (Invitrogen, Thermo Fisher Scientific, Waltham, MA, USA), 1 mM sodium pyruvate and penicillin–streptomycin (1:100) (Thermo Fisher Scientific, Waltham, MA, USA). HEK293 cells (HEK-Gs/SSTR2_HA), ectopically expressing Gαi-coupled somatostatin receptor 2 (SSTR2) GPCR as well as a luminescence cAMP probe (GloSensor-22F, Promega, Mannheim, Germany) [37], were cultivated in DMEM/F12 (containing glutamine and sodium pyruvate) supplemented with 10% heat-inactivated fetal calf serum (Invitrogen) and penicillin–streptomycin (1:100) (Thermo Fisher Scientific). Cells were grown under humidified conditions at 37 °C with 5% CO_2_, and trypsinized and reseeded every two to three days for at most 25 times. For intoxication experiments, cells were seeded in culture dishes one or two days before and treated in FCS-free media with PT and the respective compounds.

### 4.4. Sequential ADP-Ribosylation of Gαi in Lysates from Toxin-Treated Cells

CHO-K1 cells were seeded into 24-well plates and treated with PT (10 ng/mL = 0.095 nM) and defensin or H_2_O as a control in FCS-free medium for 4 h. Then, cells were washed to remove unbound protein and frozen for cell lysis. As described earlier [47], ADP-ribosylation buffer (0.1 mM Tris-HCL (pH 7.6), 20 mM DTT, 0.1 µM ATP and protease inhibitor complete (Roche)) was added and cell lysates were transferred into reaction tubes. For sequential ADP-ribosylation of Gαi in vitro, which had not been modified by PT in the cells during intoxication, recombinant PTS1 (50 nM) and biotin-labeled NAD^+^ (1 µM; R&D Systems) were added and incubated for 40 min at room temperature. Subsequently, samples were subjected to SDS-PAGE and Western blot analysis with streptavidin–peroxidase (Strep-POD, Sigma-Aldrich) for detection of biotin-labeled and thus sequentially ADP-ribosylated Gαi. Densitometric quantification of signals was performed using ImageJ software (NIH). Hsp90 (primary antibody from Santa Cruz Biotechnology, Dallas, TX, USA) signals were detected as a loading control. 

### 4.5. Cell Viability Assay

CHO-K1 cells were seeded in 96-well plates and treated with defensin or H_2_O as a control in FCS-free medium for 4 h. As a positive control, DMSO (20%) was added to the cells to cause cell death. Cell viability was determined by Cell Titer 96^®^ Aqueous One Solution cell proliferation assay (MTS assay, Promega), which was added to the cells and incubated for 60 min at 37 °C. Absorbance was measured at 490 nm via a plate reader. 

### 4.6. iGIST Bioassay

The iGIST bioassay was performed as previously described [37]. HEK293 cells ectopically expressing Gαi-coupled somatostatin receptor 2 (SSTR2) GPCR as well as a luminescence cAMP probe (GloSensor-22F, Promega) were seeded in 96-well plates with white walls and a translucent bottom (View-Plate 96, Perkin Elmer). PT (100 ng/mL) or matched buffer SolC (50% glycerol, 50 mM Tris, 10 mM glycine, 0.5 M NaCl, pH 7.5) and defensins (12 µM) or H_2_O as a control were pre-incubated in FCS-free medium for 15 minutes and then added to the cells for 5 h. Subsequently, medium was removed and 45 µL inducing medium (comprised of 2% GloSensor reagent (Promega), 400 µM of the phosphodiesterase inhibitor IBMX (Sigma) in DMEM/F12 medium and CO_2_-independent medium (4v of DMEM/F12 per 5v CO_2_-independent medium), supplemented with 0.1% (w/v) bovine serum albumin) was added and equilibrated for 45 min at room temperature in the dark. With the Orion microplate luminometer (Berthold Detection Systems) baseline luminescence was recorded for 15 min. Then cells were spiked with forskolin (10 µM, Merck Sigma, Darmstadt, Germany) to activate adenylate cyclase and octreotide acetate (20 nM, Bachem, Bubendorf, Switzerland) to activate the Gαi-coupled SSTR2 GPCR in 25 mM HEPES buffer (pH 7.4). Luminescence, corresponding to cAMP levels in the cells was measured for another 60 min. For quantification of kinetic curves, the baseline-subtracted area under the curve (AUC) was calculated by GraphPad Prism software employing the trapezoidal rule.

## Data Availability

The datasets generated and/or analyzed during the current study are either included in the study and/or available from the corresponding author on reasonable request.
